# XB130, a New Adaptor Protein, Regulates Expression of Tumor Suppressive MicroRNAs in Cancer Cells

**DOI:** 10.1371/journal.pone.0059057

**Published:** 2013-03-19

**Authors:** Hiroki Takeshita, Atsushi Shiozaki, Xiao-Hui Bai, Daisuke Iitaka, Hyunhee Kim, Burton B. Yang, Shaf Keshavjee, Mingyao Liu

**Affiliations:** 1 Division of Digestive Surgery, Department of Surgery, Kyoto Prefectural University of Medicine, Kyoto, Japan; 2 Latner Thoracic Surgery Research Laboratories, University Health Network Toronto General Research Institute, Toronto, Ontario, Canada; 3 Department of Laboratory Medicine and Pathobiology, University of Toronto, Toronto, Ontario, Canada; 4 Department of Surgery and Institute of Medical Science, Faculty of Medicine, University of Toronto, Toronto, Ontario, Canada; Hungarian Academy of Sciences, Hungary

## Abstract

XB130, a novel adaptor protein, promotes cell growth by controlling expression of many related genes. MicroRNAs (miRNAs), which are frequently mis-expressed in cancer cells, regulate expression of targeted genes. In this present study, we aimed to explore the oncogenic mechanism of XB130 through miRNAs regulation. We analyzed miRNA expression in XB130 short hairpin RNA (shRNA) stably transfected WRO thyroid cancer cells by a miRNA array assay, and 16 miRNAs were up-regulated and 22 miRNAs were down-regulated significantly in these cells, in comparison with non-transfected or negative control shRNA transfected cells. We chose three of the up-regulated miRNAs (miR-33a, miR-149 and miR-193a-3p) and validated them by real-time qRT-PCR. Ectopic overexpression of XB130 suppressed these 3 miRNAs in MRO cells, a cell line with very low expression of XB130. Furthermore, we transfected miR mimics of these 3 miRNAs into WRO cells. They negatively regulated expression of oncogenes (miR-33a: MYC, miR-149: FOSL1, miR-193a-3p: SLC7A5), by targeting their 3′ untranslated region, and reduced cell growth. Our results suggest that XB130 could promote growth of cancer cells by regulating expression of tumor suppressive miRNAs and their targeted genes.

## Introduction

Actin filament associated protein (AFAP) is a small family of adaptor proteins involved in intracellular signal transduction, cytoskeletal organization, cell motility and other cellular functions. It includes AFAP [1], AFAP1L1 (actin filament associate protein 1 like 1) [2], and XB130 (also known as actin filament associated protein 1-like 2, AFAP1L2) [3]. They have been demonstrated to participate in the regulation of various signaling pathways by forming protein-protein and/or protein-lipid complexes [1], [4], and under certain circumstances these adaptor proteins can be involved in tumorigenesis [5], [6].

XB130 is a tyrosine kinase substrate, which can be tyrosine phosphorylated by Src and other tyrosine kinases [7]–[9], and interact with Src through its N-terminal SH2 and SH3 domain binding motifs, and mediates Src related transactivation of SRE and AP-1 [7]. The N-terminus of XB130 also contains a YxxM motif that can bind to the p85α subunit of phosphatidyl inositol 3-kinase (PI3K) through its SH2 domains, and subsequently activate Akt [2], [8]. XB130 mediates cell survival and proliferation through multiple signals down-stream from Akt [9]. XB130 in human thyroid cancer cells regulates tumor growth as shown in an animal model with nude mice, through promotion of cell proliferation and inhibition of apoptosis. Moreover, knockdown of XB130 reduces expression of many genes related to cell proliferation and/or survival [10]. XB130 is also involved in the regulation of cell migration [11]. Alteration of XB130 expression has been noted in human thyroid cancer [10], esophageal cancer [12], and gastric cancer [13]. Therefore, these studies call for further examination on the function of XB130 in tumorigenesis.

MicroRNAs (miRNAs) are small non-coding RNAs (approximately 22 nucleotide lengths), which can specifically interact with the 3′-untranslated region (3′UTR) of targeted mRNAs, inhibit mRNA translation, or lead to mRNA cleavage and degradation [14]. The number of reported human miRNAs exceeds 2,000 (miRBase, Release 18 at the Sanger Institute), and miRNAs play important roles in controlling biological processes including development, differentiation, metabolism and proliferation [15]–[18]. Some miRNAs are frequently mis-expressed in cancer cells, and have recently been identified as new factors related to oncogenesis and tumor progression [19]–[22]. Several recent studies focus on the regulation of miRNA expression and function in cancer [23]–[26], including thyroid cancer [27]–[29].

Although XB130 can regulate expression of many genes related to cell proliferation [10], and promotes cell proliferation and survival via PI3K/Akt pathway [9], little is known about the mechanisms underlying its regulation of gene expression. In the present study, we sought to determine whether XB130 could regulate expression of some of these genes via down-regulation of tumor suppressive miRNAs. We examined miRNA expression level using XB130 short hairpin RNA (shRNA) stably transfected WRO thyroid cancer cells, in comparison with non-transfected cells or cells stably transfected with negative control shRNA. On the other hand, we transfected MRO cancer cells that have very low expression of XB130 with XB130 plasmid to enhance its expression. Three tumor suppressive miRNAs were identified and further studied, in terms of expression of their targeted genes, cell proliferation, and apoptosis.

## Materials and Methods

### Cell Culture

Human thyroid carcinoma WRO cells and MRO cells were from Dr. S. Asa (University of Toronto, Toronto, Canada) [30], who obtained these cells from Dr. J. Fagin (Memorial Sloan-Kettering Cancer Center, New York, NY, USA). Cells were maintained in RPMI 1640, supplemented with 10% FBS, 1 mmol/L pyruvate and nonessential amino acids (GIBCO-BRL, Gaithersburg, MD, USA), and 1% penicillin-streptomycin. We previously established WRO cells that are stably transfected with shRNA plasmids for XB130. (C3-1 and C3-4 were established from vector C3; C4-3 and C4-11 were established from vector C4) and negative control shRNA plasmid (NC1, NC9 and NC12) were established previously [10]. G418 (0.25 mg/ml) was added to culture medium of transfected cells to maintain the selection. The cells were cultured in a standard humidified incubator at 37°C with 5% CO_2_.

### Protein Studies and Western Blotting

Western blot was performed according to procedures as described previously [31], [32]. In brief, cells were lysed with modified radioimmune precipitation assay buffer (50 mmol/L Tris-HCl, pH 7.5; 150 mmol/L NaCl; 2 mmol/L EGTA; 2 mmol/L EDTA; and 1% Triton X-100) containing 10 µg/ml each of aprotinin, leupeptin, pepstatin, 1 mmol/L phenylmethylsulfonyl fluoride, 1 mmol/L Na_3_VO_4_ and 10 mmol/L NaF. Protein concentrations were measured by Pierce® BCA Protein Assay Kit (Thermo, Rockford, IL, USA).

Cell lysates containing equal amounts of total proteins were separated by SDS–PAGE and then transferred onto nitrocellulose membranes. The membranes were then probed with the indicated antibodies. Proteins were revealed by SuperSignal West Dura Extended Duration Substrate (Thermo). The intensities of protein bands were quantified using ImageJ software (http://rsb.info.nih.gov/ij/).

Monoclonal XB130 antibody was generated as described previously [7]. Antibodies for β-actin, MYC, CEBPG, FOSL1, SCL7A5, DECR1, SETD8 were purchased from Santa Cruz Biotechnology (Santa Cruz Biotechnology, CA, USA). Anti-PCNA antibody came from Abcam (Toronto, ON, Canada). Anti-Ki-67 (clone Ki-S5) came from Cedarlane (Burlington, ON, Canada).

### Microscopy and Immunofluorescence Analysis

For immunofluorescence staining, cells were grown on glass coverslips pre-treated with 50 µg/mL poly-L-lysine. Following adhesion, cells were rapidly fixed by 4% paraformaldehyde for 20 min and washed with PBS. Cells were permeabilized with 0.1% Triton-X-100 for 10 min and blocked with 1% BSA for 1 h. The coverslips were then incubated with primary antibody against XB130 for 2 h, followed by washing and incubation with secondary antibody conjugated with Alexa Fluor 594 (Invitrogen, Carlsbad, CA, USA) for 1 h. The coverslips were also stained for F-actin with Oregon Green 488 Phalloidin (Invitrogen) for 1 h and for nucleus with Hoechst dye 33342 (Invitrogen) for 10 min. Coverslips were washed with PBS and mounted on glass slides using Dako fluorescence mounting medium (Dako, Mississauga, ON, Canada). The staining was visualized using an Olympus IX81 microscope.

### MicroRNA Array and Data Analysis

Four XB130 shRNA stably transfected clones (C3-1, C3-4, C4-3 and C4-11) were used as XB130 knockdown cells. WRO cells and three negative control shRNA stably transfected clones (NC1, NC9 and NC12) were used for comparison. Total RNA was extracted using mirVana™ miRNA Isolation Kit (Ambion, Austin, TX, USA). The RNA integrity number determined by the Agilent Bioanalyzer 2100 (Agilent Technologies, Santa Clara, CA, USA) was used as a measure of the quality of the RNA. MiRNA expression profiling was performed using Human Agilent’s miRNA Microarray system (Agilent Technologies) with a SurePrint G3 8×60 K v16 platform for 1,205 human miRNAs (miRBase release 16.0). Briefly, 100 ng of total RNA were labeled with Cyanine 3-pCp and hybridized onto the arrays at 55°C for 20 h. Slides were scanned with an Agilent Technologies Scanner G2505C, and the scanned images were analyzed using Agilent Feature Extraction version 10.7.3. Differential expression analysis and hierarchical cluster analysis was performed using GeneSpring GX 11.5 (Agilent Technologies).

### Target Prediction of miRNAs

TargetScan 6.0 (http://www.targetscan.org/), microRNA.org (http://www.microrna.org/), PicTar (http://pictar.mdc-berlin.de/), and DIANA-MICROT v4/TarBase 5.0 (http://diana.cslab.ece.ntua.gr/DianaTools/) were used to search for predicted targets of miRNAs.

### Real-Time Quantitative RT-PCR for XB130

Total RNA was extracted using RNeasy kit (Qiagen, Valencia, CA, USA), and cDNA was synthesized from the total RNA using High Capacity cDNA RT kit (Applied Biosystems, Foster City, CA, USA). Quantitative RT-PCR for XB130 was performed using SYBR Green I Master PCR kit on LightCycler480 (Roche Diagnostics Corporation, Indianapolis, IN, USA) according to the manufacturer’s protocol. SDHA was used as an internal reference gene for analyses. The PCR primers for XB130 and SDHA are: XB130_forward 5′-AGCACAGCACTGGTGAAGAA-3′, XB130_reverse 5′-GTTGCTTGTTGATGGTCACT-3′, SDHA_forward 5′-CGGCATTCCCACCAACTAC-3′ and SDHA_reverse 5′-GGCCGGGCACAATCTG-3′. Expression of XB130 was normalized using the 2-ΔΔCT method relative to SDHA. The ΔCt was calculated by subtracting the Ct values of SDHA from the Ct values of the XB130. Fold change in the gene was calculated by the equation 2-ΔCt [33]. All assays were performed in triplicate. The mean of expression of XB130/SDHA ratio in WRO cells was set to 1 and the results were expressed as mean and standard deviation of fold of XB130 changes.

### Real-Time Quantitative RT-PCR for pri-miRNAs and Mature miRNAs

To quantify expression of the pri-miRNAs and mature miRNAs, total RNA was extracted using mirVana™ miRNA Isolation Kit (Ambion). As for quantifications of the pri-miRNAs, the cDNA was synthesized using High Capacity cDNA RT kit (Applied Biosystems). Real-time PCR reactions were performed using a TaqMan® Universal Master Mix II and the TaqMan® pri-miRNA Assays specific for has-mir-33a, has-mir-149 and has-mir-193a-3p on a 7900 Real-time PCR system (Applied Biosystems).

As for quantifications of the mature miRNAs, a TaqMan® MicroRNA RT Kit and TaqMan® MicroRNA assays specific for hsa-miR-33a, has-miR-149 and has-miR-193a-3p (Applied Biosystems) were used for RT reactions. Real-time PCR reactions were performed using a TaqMan® Universal Master Mix II and the TaqMan® MicroRNA assays on a 7900 Real-time PCR system (Applied Biosystems). In both quantifications, RUN6B (U6) was assessed as endogenous control, and the results was normalized using the 2-ΔΔCT method same as real-time qRT-PCR for XB130.

### Cell Transfection

Generation of plasmid of GFP-alone, GFP-tagged XB130 (GFP-XB130) and its N-terminus deletion mutant (GFP-XB130ΔN) were described previously [11]. MRO cells were transiently transfected using 60 µL Lipofectamine 2000 (Invitrogen) on a 100 mm dish as described in the manufacturer’s instructions. The medium containing plasmid was replaced with fresh medium 24 h after the transfection, and GFP positive cells were isolated by fluorescence activated cell sorting (FACS) Aria II (BD Biosciences, San Jose, CA) 48 hrs after the transfection. GFP positive cells were then used for western blotting and cell proliferation assays.

mirVana™ miRNA Mimic of miR-33a, miR-149 and miR-193a and mirVana™ miRNA Mimic Negative Control #1 were obtained commercially (Ambion). WRO cells were transfected with 50 pmol miR mimic using 5 µL Lipofectamine 2000 (Invitrogen) on a 6-well plate according to the manufacturer’s protocol. The medium containing plasmid was replaced with fresh medium 24 h after the transfection and total RNA and protein were extracted 48 h after the transfection.

### Luciferase Reporter Assay

Seed sequences of miR-33a, miR-149 and miR-193a-3p and pairing 3′UTR sequences of MYC, FOSL1 and SLC7A5 were predicted by TargetScan. 3′UTR sequences were cloned downstream to firefly luciferase of pmirGLO Dual-Luciferase miRNA Target Expression Vector and verified by sequencing (Promega, Madison, WI, USA). The pmirGLO construct and the miR mimic or control miR mimic were co-transfected into WRO cells cultured in 96-well plates using Lipofectamine 2000 (Invitrogen) according to the manufacturer’s protocol. Forty-eight hours after transfection, firefly and Renilla luciferase activities were measured using a Dual Luciferase Reporter Assay System (Promega). Firefly luciferase was normalized to Renilla luciferase activity. The mean of luciferase activity of Firefly/Renilla ratio in WRO cells co-transfected with each of the pmirGLO construct and control miR mimic was set to 1. Data were expressed as mean and standard deviation of five independent experiments.

### Cell Proliferation and Apoptosis Assays

WRO cells were plated in quintuple wells of 96-well microtiter plates (100 µl/well) for cell proliferation assay, in 12-well microtiter plates (1 ml/well) for cell count assay, and in 6-well microtiter plates (2 ml/well) for apoptosis assay, at 5×10^4^ cells/ml and cultured for 24 h. The cells were then transfected with miRNA mimic as described above.

Cell proliferation was evaluated using CellTiter 96® Aqueous One Solution Cell Proliferation Assay (Promega) at each time point (0, 24, 48, and 72 h) after the transfection. After replaced with fresh medium, 20 µl of the CellTiter 96® AQueous One Solution Reagent was added into each well and the plates were incubated at 37°C for 2 h in a humidified, 5% CO_2_ atmosphere. The absorbance of samples was read at 490 nm with an automatic plate reader (Thermo). The background absorbance of medium-alone was subtracted. The proliferation index was calculated as the mean absorbance of cells at the indicated time point divided by the mean absorbance of cells at 0 h after transfection and expressed as mean and standard deviation. In cell count assay, cells were detached from the flasks with a trypsin-EDTA solution and counted by a hemocytometer at each time point. The cell number was expressed as mean and standard deviation.

In apoptosis assay, the transfected cells were harvested 72 h after the transfection and stained with fluorescein isothiocyanate-conjugated annexin V and phosphatidylinositol (PI), using ANNEXIN V – FITC Kit (Beckman Coulter, Brea, CA, USA) according to the manufacturer’s protocols and analyzed by BD AccuriTM C6 Flow Cytometer (BD Biosciences, San Jose, CA).

## Results

### Characterization of XB130 shRNA Stably Transfected WRO Cells

**Figure 1:**
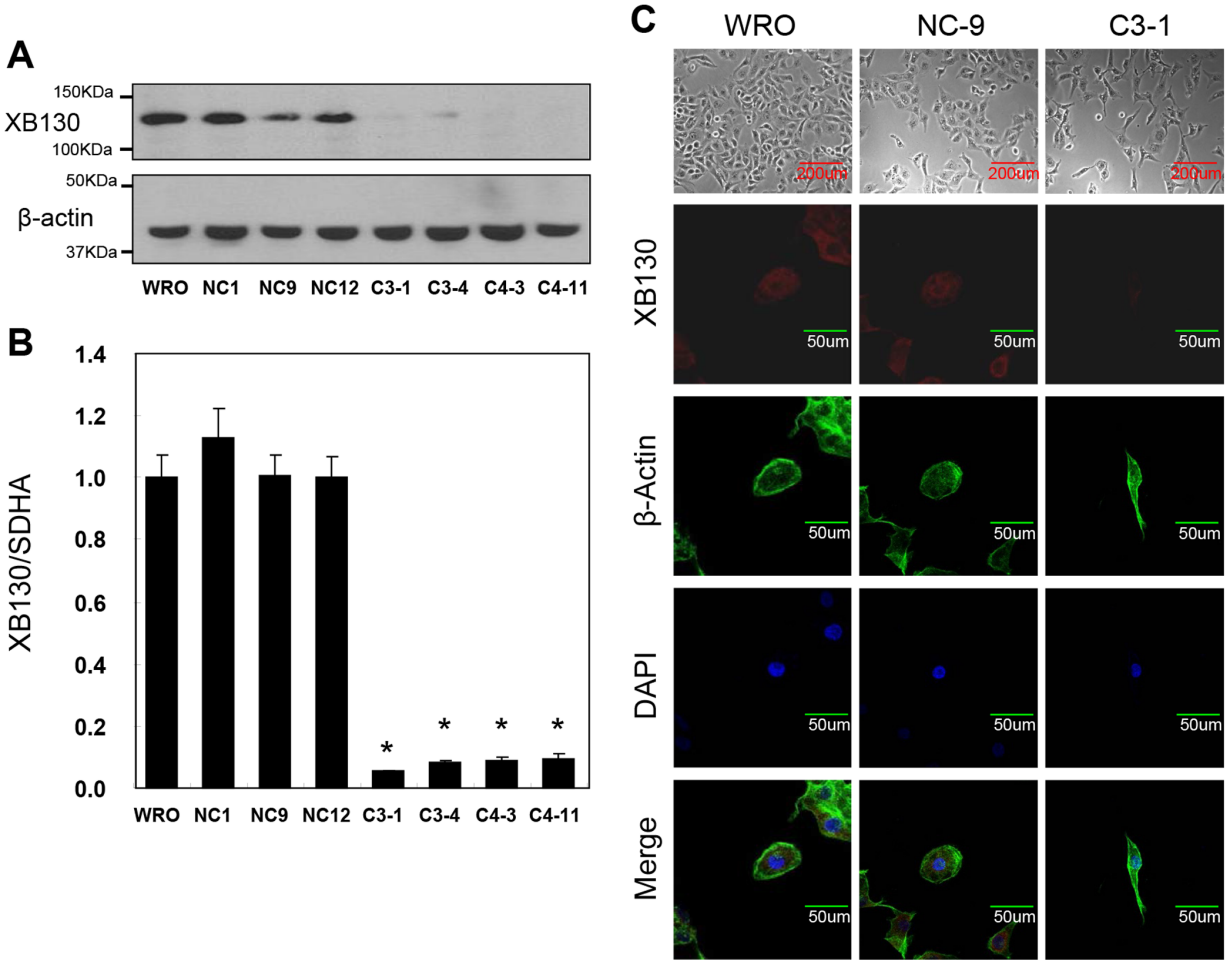
Decreased XB130 expression in XB130 shRNA stably transfected WRO cells. The following cells were examined: WRO cells (WRO), negative control shRNA transfected WRO cells (NC1, NC9 and NC12) and XB130 shRNA transfected WRO cells (C3-1, C3-4, C4-3 and C4-11). (**A**) XB130 shRNA effectively reduced XB130 protein levels. (**B**) Real-time quantitative RT-PCR revealed that the expression of XB130 mRNA in XB130 shRNA transfected WRO cells were significantly lower than WRO cells (*P<0.05). (**C**) WRO, NC9 and C3-1 were immune-stained with an XB130 antibody and counterstained for F-actin and nuclei with Oregon Green 488 Phalloidin and Hoechst 33342. The expression of XB130 protein in cytoplasm disappeared in the XB130 shRNA transfected cells.

To study the roles of XB130 in regulating WRO cell activities, we stably transfected the cells with shRNA targeting XB130. Expression of XB130 was significantly lower in these clones (C3-1, C3-4, C4-3 and C4-11) than that in non-transfected WRO cells and other three clones stably transfected with a negative control shRNA (NC1, NC9, NC12), as determined by Western blot (Fig. 1A) and by real-time quantitative RT-PCR (Fig. 1B). The expression of SDHA mRNA was used as a housekeeping gene for qRT-PCR (Fig. 1B), because its expression does not change under different experimental conditions [10], [34], [35]. Interestingly, XB130 knockdown cells showed elongated morphology when examined with phase-contrast microscopy. In addition, immunofluorecence staining revealed that the expression of XB130 in C3-1, C3-4, C4-3 and C4-11 cells was barely detectable, and F-actin staining also showed elongated morphology at single cell levels (Fig. 1C, Fig. S1).

### MicroRNA Expression Profile in XB130 shRNA Transfected Cells

To identify miRNAs regulated by XB130, we performed miRNA expression profile assay, by comparing XB130 shRNA stably transfected WRO cells (C3-1, C3-4, C4-3 and C4-11) with controls (WRO, NC1, NC9 and NC12) using a microRNA array assay. GeneSpring GX analysis showed that 38 miRNAs were significantly changed by the stable knockdown of XB130 in WRO cells (p value <0.05), of which, 16 were up-regulated (Table 1) and 22 were down-regulated (Table S1) in XB130 shRNA transfected cells.

**Table 1:** Significantly upregulated miRNAs in XB130 shRNA transfected Cells.

miRNA name	Fold change	p-value
hsa-miR-33a	2.994	0.003
hsa-miR-191*	2.435	0.043
hsa-miR-181a*	2.399	0.030
hsa-miR-940	2.289	0.034
hsa-miR-30e*	2.277	0.026
hsa-miR-193a-3p	2.124	0.005
hsa-miR-21	2.123	0.005
hsa-miR-210	2.089	0.041
hsa-miR-149	2.075	0.023
hsa-miR-17*	1.892	0.035
hsa-let-7f-1*	1.861	0.043
hsa-miR-23a	1.688	0.010
hsa-miR-27a	1.637	0.010
hsa-miR-365	1.567	0.028
hsa-miR-4284	1.428	0.017
hsa-miR-30d	1.186	0.032

Note: Two strands come from one stem loop precursor miRNA (pre-microRNA) by dicer processing. One of them is indicated with an asterisk. For example, has-miR-191* and has-miR-191 are two sequences from the same precursor.

**Figure 2:**
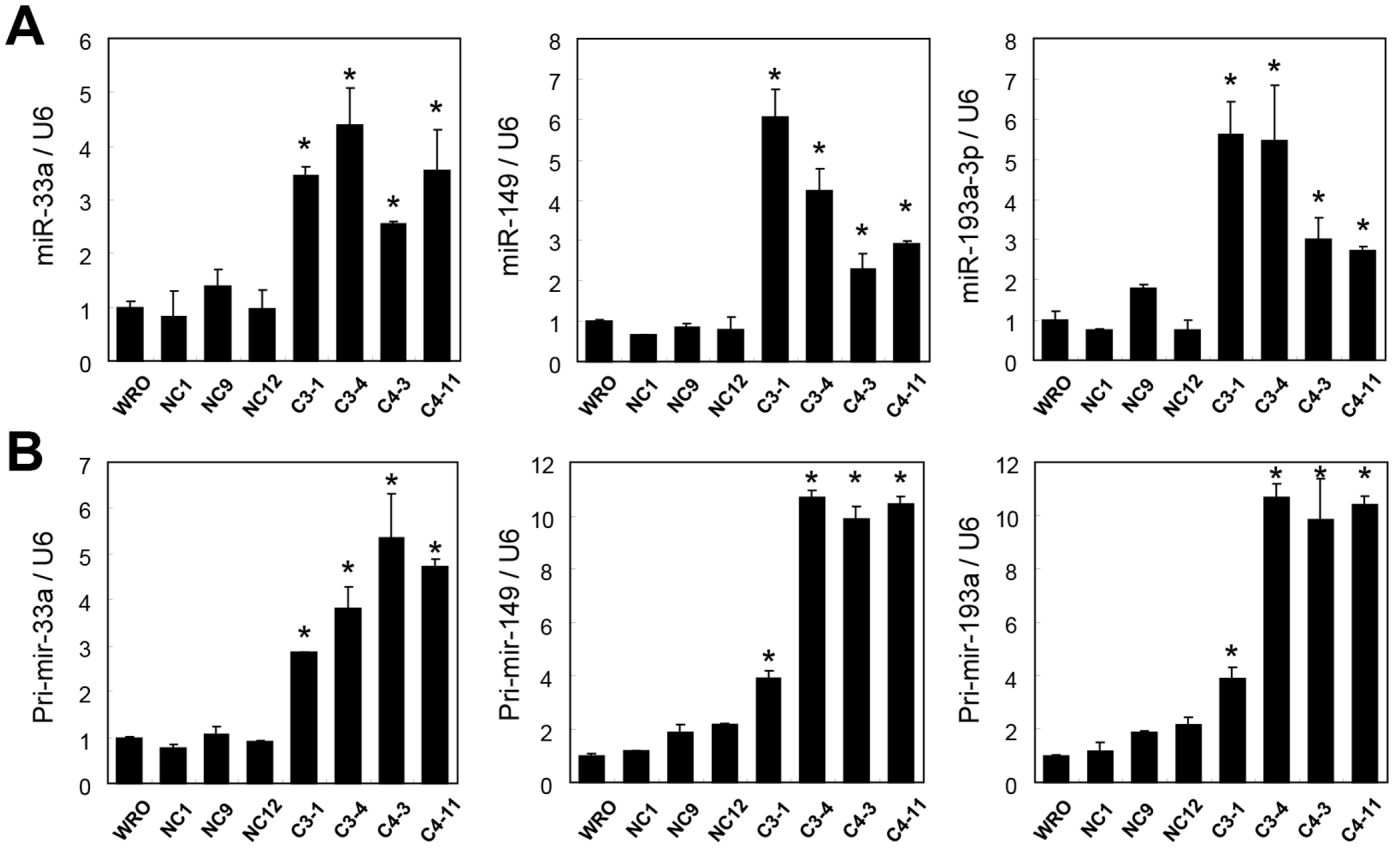
XB130 shRNA transfected WRO cells showed increased levels of miR-33a, miR-149 and miR-193a-3p by real-time quantitative RT-PCR. The mature forms (**A**) and primary transcripts (**B**) of miR-33a, miR-149 and miR-193a-3p are significantly higher in XB130 shRNA transfected WRO cells (C3-1, C3-4, C4-3 and C4-11) than in WRO (*P<0.05).

To determine the mechanisms by which XB130 regulates cell proliferation, we focused on miRNAs repressed by XB130 and selected miR-33a, miR-193a-3p and miR-149 for further analysis, which have been reported to show tumor suppressor functions in various cancers [36]–[38]. To verify the effects of XB130 on miR-33a, miR-149 and miR-193a-3p, we quantified levels of these 3 miRNAs using real-time qRT–PCR. Both the mature miRNA (Fig. 2A) and pri-miRNA (Fig. 2B) levels of these three miRNAs in XB130 shRNA stably transfected WRO cells (C3-1, C3-4, C4-3 and C4-11) were significantly higher than those in the non-transfected WRO cells or cells stably transfected with negative control shRNA (NC1, NC9 and NC12).

### Ectopic XB130 Expression Suppressed Primary and Mature Forms of miR-33a, miR-149 and miR-193a-3p

**Figure 3:**
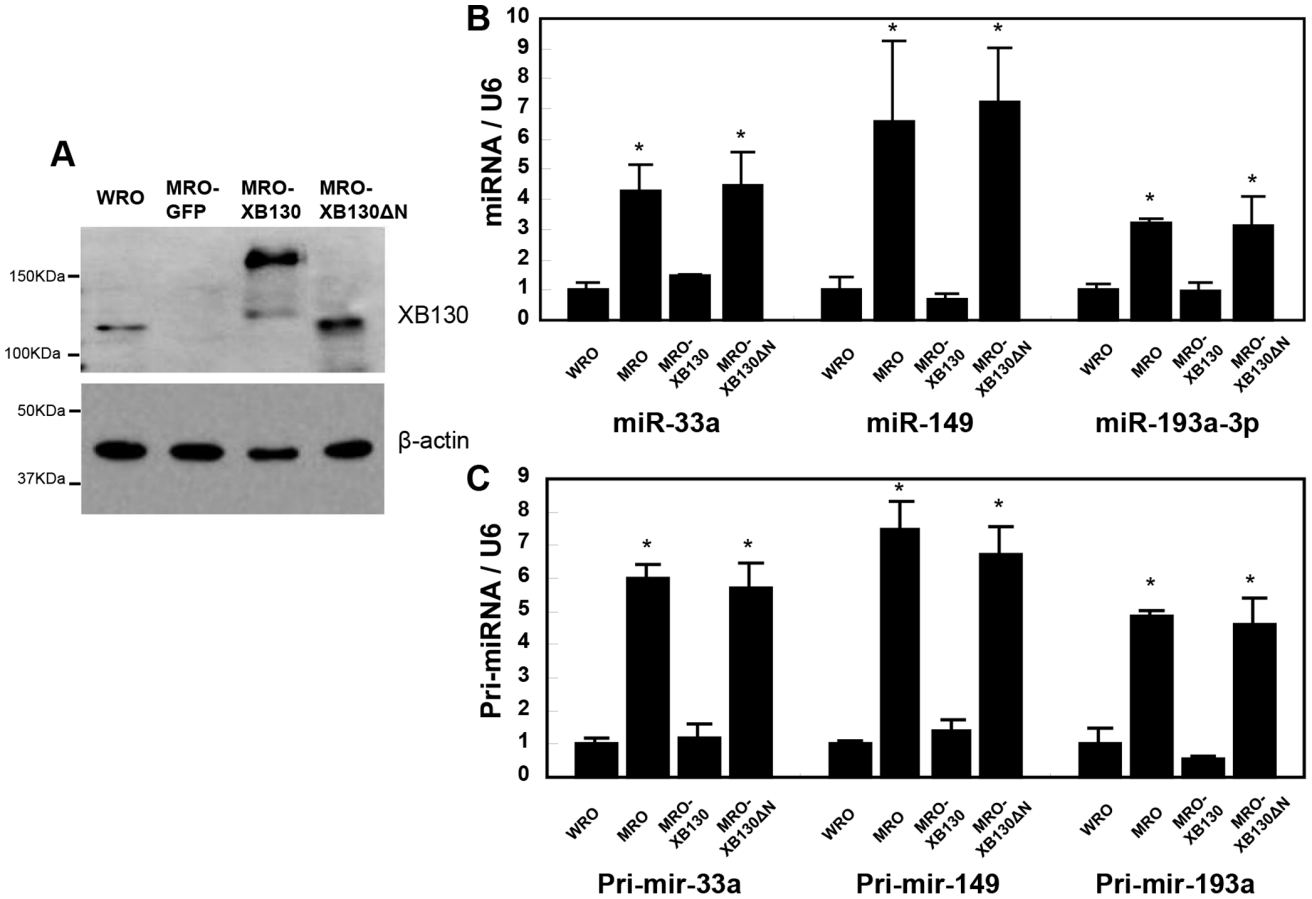
Ectopic XB130 expression in MRO cells reduced levels of miR-33a, miR-149 and miR-193a-3p. MRO cells were transfected with GFP vector alone, or GFP-XB130, GFP-XB130ΔN (XB130 N-terminus deletion mutant). GFP positive cells were collected by FACS. (**A**) Western blotting revealed that MRO cells express very low XB130. Transfection of GFP-XB130 (MRO-XB130) or GFP-XB130ΔN (MRO-XB130ΔN) showed XB130 expression in MRO cells. The mature forms (**B**) and primary transcripts (**C**) of miR-33a, miR-149 and miR-193a-3p were examined by real-time quantitative RT-PCR. These miRNAs and pri-miRNAs in MRO cells were significantly higher than that in WRO cells, and significantly reduced by overexpression of GFP-XB130, but not by GFP-XB130ΔN. *P<0.05 compared with WRO cells.

To further confirm the regulatory effects of XB130 on expression of miR-33a, miR-149, and miR-193a-3p, we performed a “rescue” study on MRO cells, which have very low XB130 expression as determined by western blotting. We transfected MRO cells with plasmids expressing GFP-alone, GFP-XB130, or its N-terminus deletion mutant, GFP-XB130ΔN. GFP positive cells were collected by FACS, and the expression of GFP-XB130 and GFP-XB130ΔN proteins in transfected MRO cells was demonstrated by western blotting (Fig. 3A). In contrast to WRO cells, MRO cells showed significantly higher expression levels of miR-33a, miR-149 and miR-193a-3p, both the mature and primary forms. XB130-GFP overexpression resulted in a significant down-regulation of both mature miRNAs and pri-miRNAs, whereas overexpression of GFP-XB130ΔN did not elicit such effects (Fig. 3B and C). Since the N-terminus of XB130 contains functional motifs that can interact with Src [7] and p85α subunit of PI3K [8], the lack of effects of this mutant suggests that the “rescue” effects of GFP-XB130 may be related to Src and/or PI3K related mechanisms. The fact that both the mature and pri-miRNA levels were regulated similarly by XB130 suggested that XB130 affected these three miRNAs at least partially at the transcriptional level.

### Overexpression of miR-33a, miR-149 and miR-193a-3p Reduced Down-stream Targeted Proteins Related to Cell Proliferation

**Figure 4:**
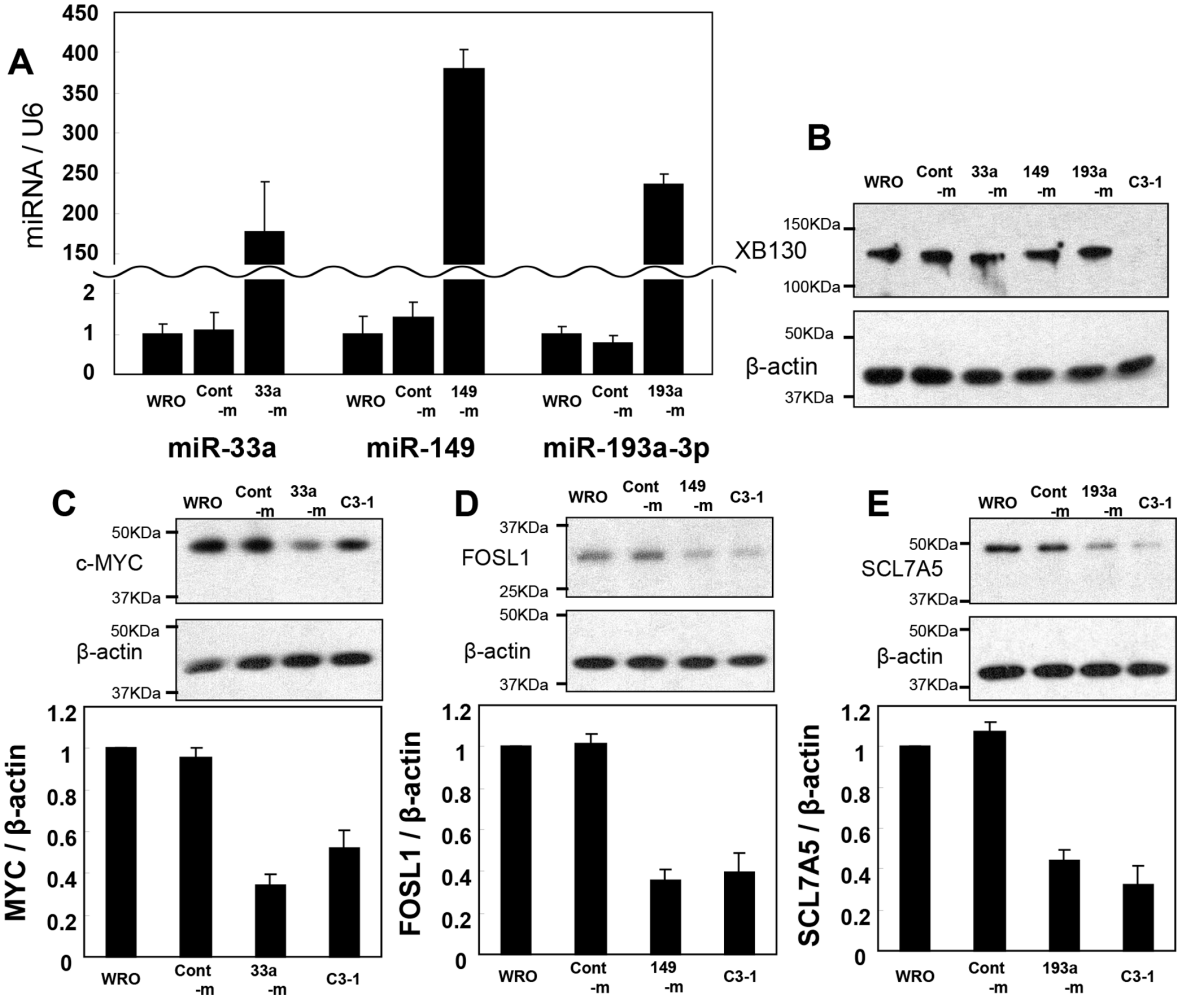
Overexpression of miR-33a, miR-149 and miR-193a-3p mimic reduced expression levels of predicted target proteins in WRO cells. (**A**) Transfection of miR mimics (33a-m, 149-m and 193a-m) significantly increased respective miRNA expression, whereas control miR mimic (cont-m) had no such effect, as quantified by real-time quantitative RT-PCR. (**B**) Western blotting shows that transfection of miR mimic did not change XB130 protein levels. (**C–E**) MYC, FOSL1 and SCL7A5 are predicted target of miR-33a, miR-149 and miR-193-3p, respectively. Upper panel shows examples that the expression of the predicted target protein was decreased by respective miR mimic transfections as determined by Western blotting. Lower panel shows the quantification of the expression levels by densitometry.

We previously reported that in XB130 shRNA stably transfected WRO cells, 57 genes related to cell proliferation or survival were significantly altered, and most of these genes were down-regulated by XB130 shRNA [10]. We speculated that among these genes, some are targets of miR-33a, miR-149 and miR-193a-3p. Using TargetScan 6.0, microRNA.org, PicTar, and DIANA-MICROT v4/TarBase 5.0, we identified MYC and CEBPG as candidates of target genes for miR-33a, CEBPG and FOSL1 for miR-149, SLC7A5, DECR1 and SETD8 for miR-193a-3p. To evaluate whether these miRNAs actually regulate expression of candidate targets, we transfected miR mimic into WRO cells. Transfection of these three miR mimics significantly increased levels of respective miRNAs (Fig. 4A), but did not affect XB130 protein levels (Fig. 4B). In comparison with WRO cells, the protein levels of MYC, FOSL1 and SLC7A5 were lower in XB130 shRNA stably transfected cells (C3-1 as an examples) (Fig. 4C–E). Overexpression of miR-33a resulted in a decrease of MYC protein levels (Fig. 4C). Similarly, overexpression of miR-149 down-regulated FOSL1 protein levels (Fig. 4D), and overexpression of miR-193-3p down-regulated SCL7A5 protein levels (Fig. 4E). The negative control mimic (Cont-m) did not affect these three proteins (Fig. 4C–E). Protein levels of CEBPG, DECR1 and SETD8 were not changed by overexpression of these miR mimics (data not shown).

Since over-expression of GFP-XB130 in MRO cells suppressed expression of miR-33a, miR-149, and miR-193a-3p (Fig. 3A–3B), we also tested whether it could subsequently affect their down-stream targeted proteins. Western blots showed that in comparison with GFP-alone transfected MRO cells, GFP-XB130 transfected cells showed higher MYC, FOSL1 and SLC7A5. In comparison with GFP-XB130ΔN transfected cells, the FOSL1 and SLC7A5 levels were also clearly higher in GFP-XB130 transfected cells (Fig. S2). These results supported the miRNA mimic data in WRO cells.

### MiR-33a, miR-149 and miR-193a-3p Directly Targeted Binding Site in the 3′UTR of MYC, FOSL1 and SLC7A5, Respectively

**Figure 5:**
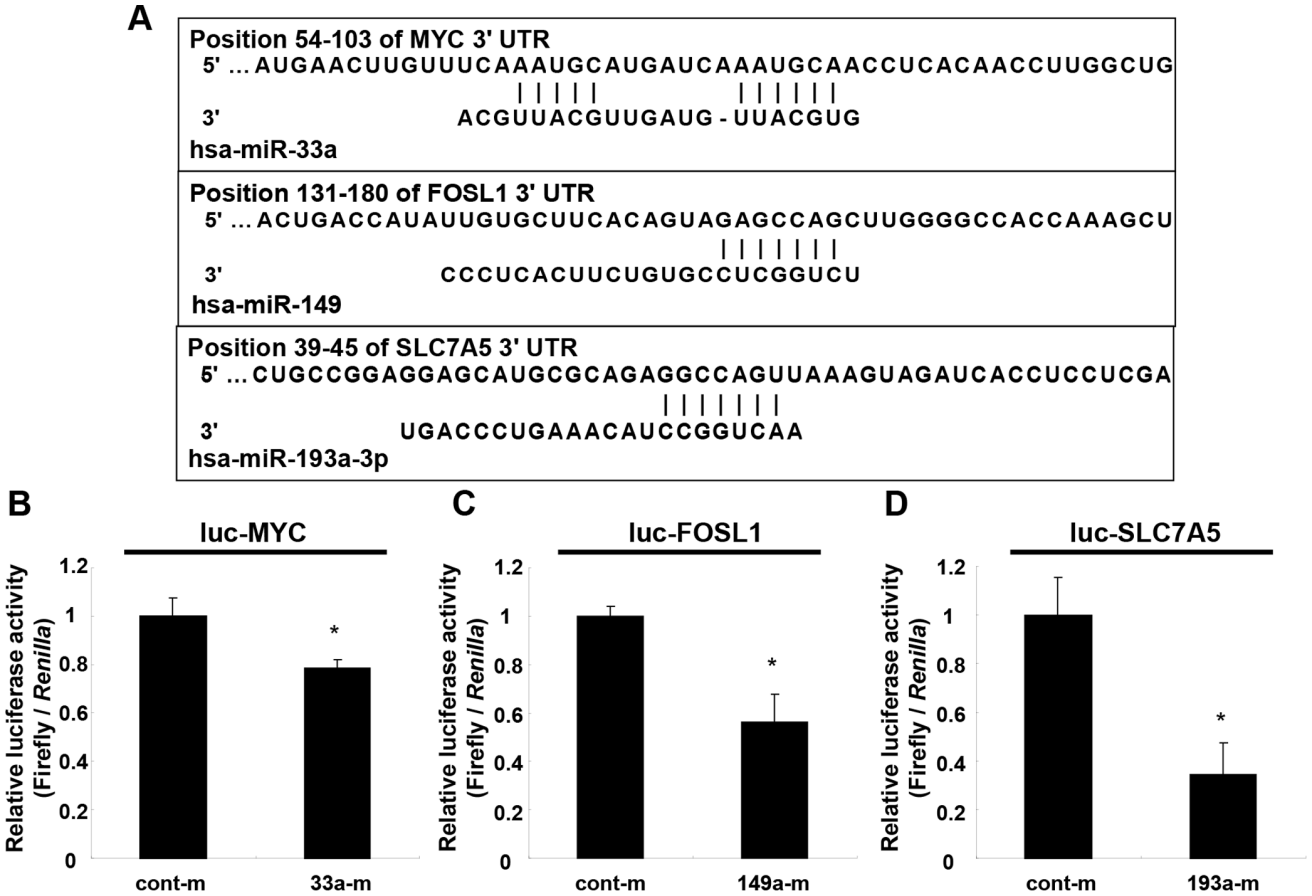
MiR-33a, miR-149 and miR-193a-3p directly target a potential binding site in the 3′UTR of MYC, FOSL1 and SLC7A5, respectively. (**A**) Seed sequences of miR-33a, miR-149 and miR-193a-3p and pairing 3′UTRs sequence of MYC, FOSL1 and SLC7A5 as predicted by TargetScan are shown. The pmirGLO construct with the cloned 3′UTR sequences (luc-MYC, luc-FOSL1 and luc-SLC7A5) and the miR mimic (33a-m, 149-m and 193a-m) or control miR mimic (cont-m) were co-transfected into WRO cells. (**B**) Overexpression of miR-33a significantly reduced luciferase activity of the pmirGLO construct with cloned 3′UTR sequences of MYC (luc-MYC). Similarly, overexpression of miR-149 and miR-193-3p significantly reduced luciferase activity of the luc-FOSL1 (**C**) and the luc-SCL7A5 (**D**), respectively.

We then used the pmirGLO Dual-Luciferase reporter system to determine whether the reduction in MYC, FOSL1 and SLC7A5 levels by overexpression of miR-33a, miR-149 and miR-193a-3p miR mimic was through the 3′UTR of their targeted mRNAs. A considerable complementarity was identified, using the algorithms in TargetScan between sequences within the seed regions of miR-33a, miR-149 and miR-193a-3p and sequences in the 3′UTR of MYC, FOSL1 and SLC7A5, respectively (Fig. 5A). The pmirGLO constructs that contain a luciferase reporter and the 3′UTR of each of these 3 genes were generated. WRO cells were co-transfected with the pmirGLO constructs and each of the miR mimics or a negative control miR. Overexpression of miR-33a significantly reduced luciferase activity of the pmirGLO construct with cloned 3′UTR sequences of MYC (Fig. 5B). Similarly, overexpression of miR-149 and miR-193-3p significantly reduced luciferase activities when the cells were transfected with the luciferase construct containing the 3′UTRs of FOSL1 and SCL7A5 (Fig. 5C and D). These results indicated that these 3 miRNAs might affect the expression of related target genes by binding to their 3′UTRs.

### Reduced Cell Proliferation by Overexpression of miR-33a, miR-149 and miR-193a-3p

**Figure 6:**
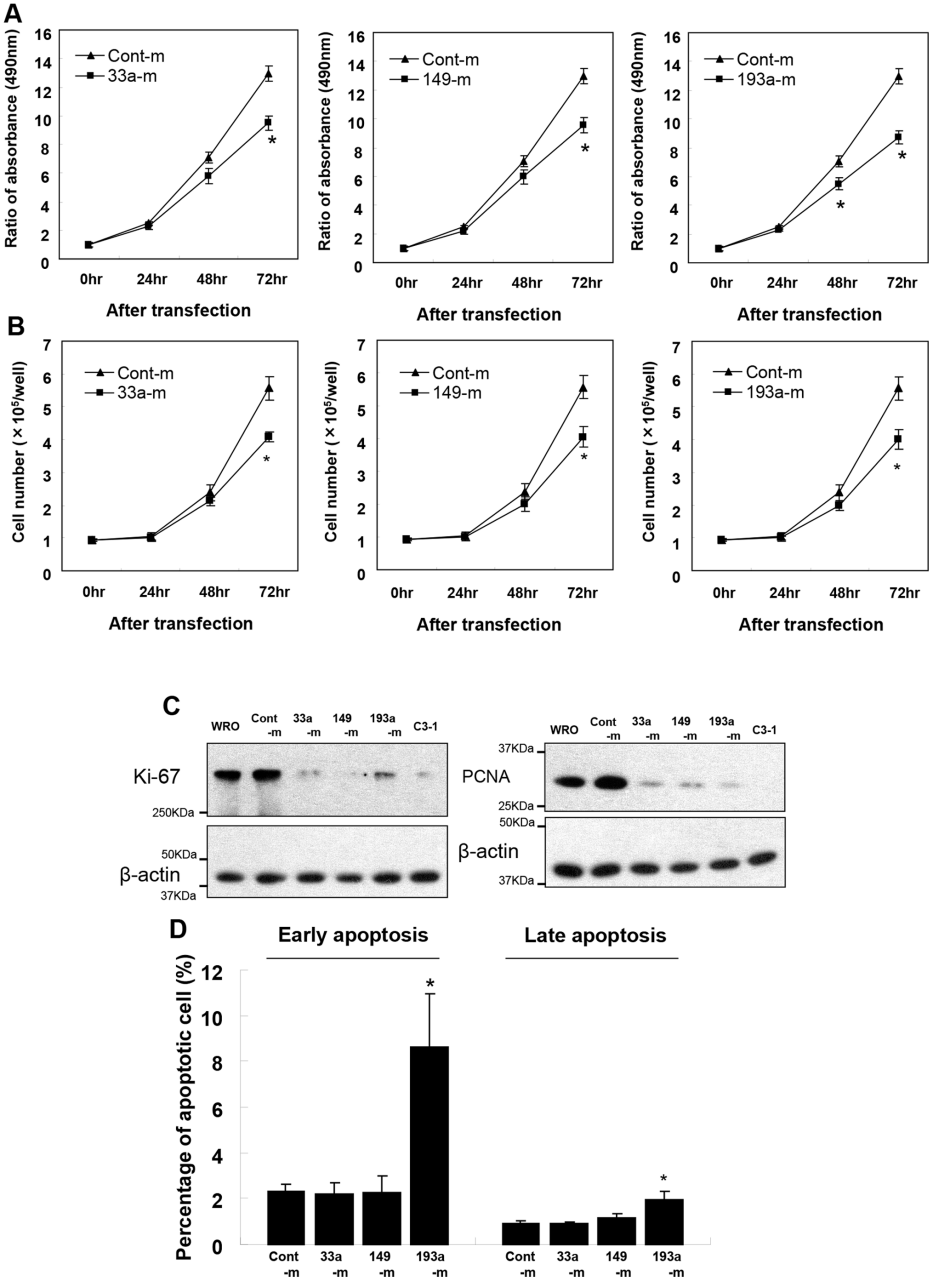
Suppressive effects of miR-33a, miR-149 and miR-193a-3p on proliferation in WRO cells. The numbers of viable cells were assessed by MTS assay (A) and cell counting (B) at 24, 48, and 72 h post-transfection of control mimic (cont-m) and specific miR mimic (33a-m, 149-m and 193a-m). The overexpression of miR-33a, miR-149 and miR-193a-3p led to reduction in cell proliferation. (**C**) Cell proliferation markers, Ki-67 and PCNA levels, were also reduced in miR mimic treated cells (33a-m, 149-m and 193a-3p). (**D**) Apoptosis was determined by flow cytometry using PI/annexin V double staining. Overexpression of miR-193a-3a significantly induced both early apoptosis (annexin V positive/PI negative) and late apoptosis (annexin V/PI double positive) in WRO cells. *: P<0.05.

MYC, FOSL1 and SLC7A5 are known to participate in the regulation of cell proliferation. Since transfection of each of these miR mimics down-regulated respective targeted protein, we then examined the effects of these miR mimics on cell proliferation. Proliferation of WRO cells was decreased by the transfection of miR mimic of miR-33a, miR-149 or miR-193a-3p, as determined by MTT assay (Fig. 6A), or by cell counting (Fig. 6B), in comparison with control miR mimic transfected cells at 72 h after cell seeding. Additionally, the expression of cell proliferation markers, Ki-67 and PCNA, were reduced by each of these three miR mimics in comparison with non-transfected WRO cells or cells transfected with control mimic (Fig. 6C). On the other hand, overexpression of GFP-XB130, and to less extend of GFP-XB130ΔN increased Ki67 expression in MRO cells (Fig. S2).

To determine whether the reduced cell number was due to apoptosis, we performed flow cytometry with PI/Annexin V double staining. Annexin V positive cells represent early apoptosis, while Annexin V and PI double positive cells represent late apoptosis [10]. Overexpression of miR-193a-3a (but not other two miRNAs) significantly induced both early and late apoptosis in WRO cells (Fig. 6D). These results suggest that the reduced cell numbers in miR-33a and miR-149 mimics are mainly due to inhibition on cell proliferation, whereas miR-193a-3p may reduce cell proliferation and induce apoptosis.

## Discussion

In our previous study, microarray analysis identified 246 genes significantly changed by stably knock down of XB130 in WRO thyroid cancer cells. Especially, 57 out of the 246 genes are related to cell proliferation or survival, including many transcription regulators [10]. It has been estimated that approximately 30% of all human genes can be regulated by miRNAs [39]. The alterations in the miRNA processing contributes to tumorigenesis [40]. Therefore, we hypothesized that XB130 may regulate expression of growth-related miRNAs and subsequently control genes related to cell proliferation and survival. To evaluate this hypothesis, we analyzed miRNA expression in XB130 knockdown WRO cells. MicroRNA array analysis data showed that 38 miRNAs were differently expressed in XB130 knockdown cells. Among them, we focused on miR-33a, miR-149 and miR-193a-3p that have been reported as tumor suppressive miRNAs in various cancers. MiR-33a decelerates cell proliferation acting through inhibition of proto-oncogene Pim-1 in lymphoma and colon cancer cells [36]. miR-149 expression has a direct correlation with KCNMA1 and LOX oncogenes in clear cell renal cell carcinoma [37]. Similarly, miR-193-3p targets JNK1 and inhibits cell-cycle progression and proliferation in breast cancer [38]. We validated up-regulation of miR-33a, miR-149 and miR-193a-3p in XB130 knockdown cells by real-time qRT-PCR.

In microRNA expression processes, RNA polymerase II produces long primary miRNA transcripts called pri-miRNAs, which are cropped and cleaved by a multi-protein complex including DROSHA and DICER1 to produce mature functional miRNAs [41]. How the miRNA expression is regulated is still unclear, yet various mechanisms have been suggested. It has been shown that p53 was responsible for the post-transcriptional maturation of miR-16-1, miR-143 and miR-145, whereas expression of related pri-miRNAs was not influenced by p53 [25]. On the other hand, BRCA1 controls miR-155 expression by regulating a transcription mechanism [24]. In the present study, the expression of pri-mir-33a, miR-149 and miR-193a-3p was also increased in XB130 knockdown cells, suggesting that XB130 may affect the transcription of these pri-miRNAs and subsequently affect the levels of mature miRNAs.

We also over-expressed XB130 in MRO thyroid cancer cells, which have very low levels of XB130. Schweppe et al. have found cross-contamination among commonly used thyroid cancer cell lines. MRO cells used in that study was suspected to be an HT-29 colon cancer derived cell line [42]. Our results need to be interpreted with cautions. Nonetheless, overexpression of XB130 significantly reduced expression of these 3 miRNAs, both mature miRNAs and pri-miRNAs. Moreover, this regulatory effect was inhibited by deletion of the N-terminus of XB130. The N-terminal region of XB130 includes several tyrosine phosphorylation sites and a proline-rich sequence that could interact with Src homology 2 and 3 domain-containing proteins, such as Src and p85α subunit of PI3K [3]. These data indicate that the regulation of these miRNAs by XB130 may be mediated trough specific signal transduction pathways.

We also examined the functions of these miRNAs on regulation of their predicted target genes and on cell proliferation in WRO cells. Firstly, overexpressed mimics of these miRNAs reduced the expression of their predicted target protein. Moreover, these miRNAs may directly bind to 3′UTR of targeted genes, as shown by the luciferase reporter assays. Interestingly, all three genes whose protein levels are down-regulated by overexpression of related miRNAs are known as oncogene. MYC, c-Myc-encoded protein, functions in cell proliferation and differentiation in several types of human cancers, including lung, breast, colon and thyroid cancers [43], [44]. FOSL1 has proto-oncogene function by dimerizing with proteins of the Jun family to form AP-1 complex, a transcription factor that controls critical cellular processes including differentiation, proliferation, and apoptosis [45], [46]. Kim et al., have reported thyroid malignant tumors have higher expression of FOSL1 (also called Fra-1) than in benign tumors [47]. SLC7A5 (also called LAT1) is overexpressed in many cancer types, and its expression levels are usually correlated to cancer progression, aggressiveness and prognosis [48], [49]. SLC7A5 siRNA significantly reduced proliferation of small cell lung cancer cells [50] and KB human oral cancer cells [51]. Indeed, overexpression of miR-33a, miR-149 and miR-193a-3p resulted in inhibition of cell growth. Taken together, our results support tumor-suppressive functions for miR-33a, miR-149 and miR-193a-3p through down-regulation of related oncogene.

In summary, this study demonstrates a novel pro-oncogenic mechanism of XB130, regulating oncogenes through tumor suppressive miRNAs. It is plausible that some of the miRNAs decreased by XB130 knockdown might suppress other target genes (such as cell cycle inhibitors). miRNAs have been proposed as therapeutic targets for cancers [52]. Therefore, how XB130 expression controls miRNA transcription and processing, and how XB130 related miRNA affects down-stream proteins, and consequently affect cell proliferation and survival may provide new knowledge in cancer research.

## Supporting Information

Figure S1 **Immunofluorecence staining for XB130.** As reported in Fig. 1, the other two negative control shRNA transfected WRO cell lines (NC1 and NC12) and three XB130 shRNA transfected WRO cell lines (C3-4, C4-3 and C4-11) were immune-stained with the XB130 antibody. The expression of XB130 protein significantly decreased in XB130 shRNA transfected cells.
(TIF)

Figure S2 **Ectopic XB130 expression in MRO cells increased levels of MYC, FOSL1, SCL7A5 and Ki67.** MRO cells were transfected with GFP vector alone, or GFP-XB130, GFP-XB130ΔN (XB130 N-terminus deletion mutant). GFP positive cells were collected by FACS. Western blotting revealed higher levels of MYC, FOSL1, SCL7A5 and Ki67, in comparison to GFP-alone transfected cells. The MYC and Ki67 levels in GFP-XB130ΔN transfected cells were also higher than that in GFP-alone cells.
(TIF)

Table S1 **Significantly downregulated miRNAs in XB130 shRNA transfected cells.**
(DOC)
