# Supplementary figures and images for: XB130, a New Adaptor Protein, Regulates Expression of Tumor Suppressive MicroRNAs in Cancer Cells
Source: PLoS One. 2013 Mar 19;8(3):e59057. doi: 10.1371/journal.pone.0059057 (PMC3602428; doi:10.1371/journal.pone.0059057)

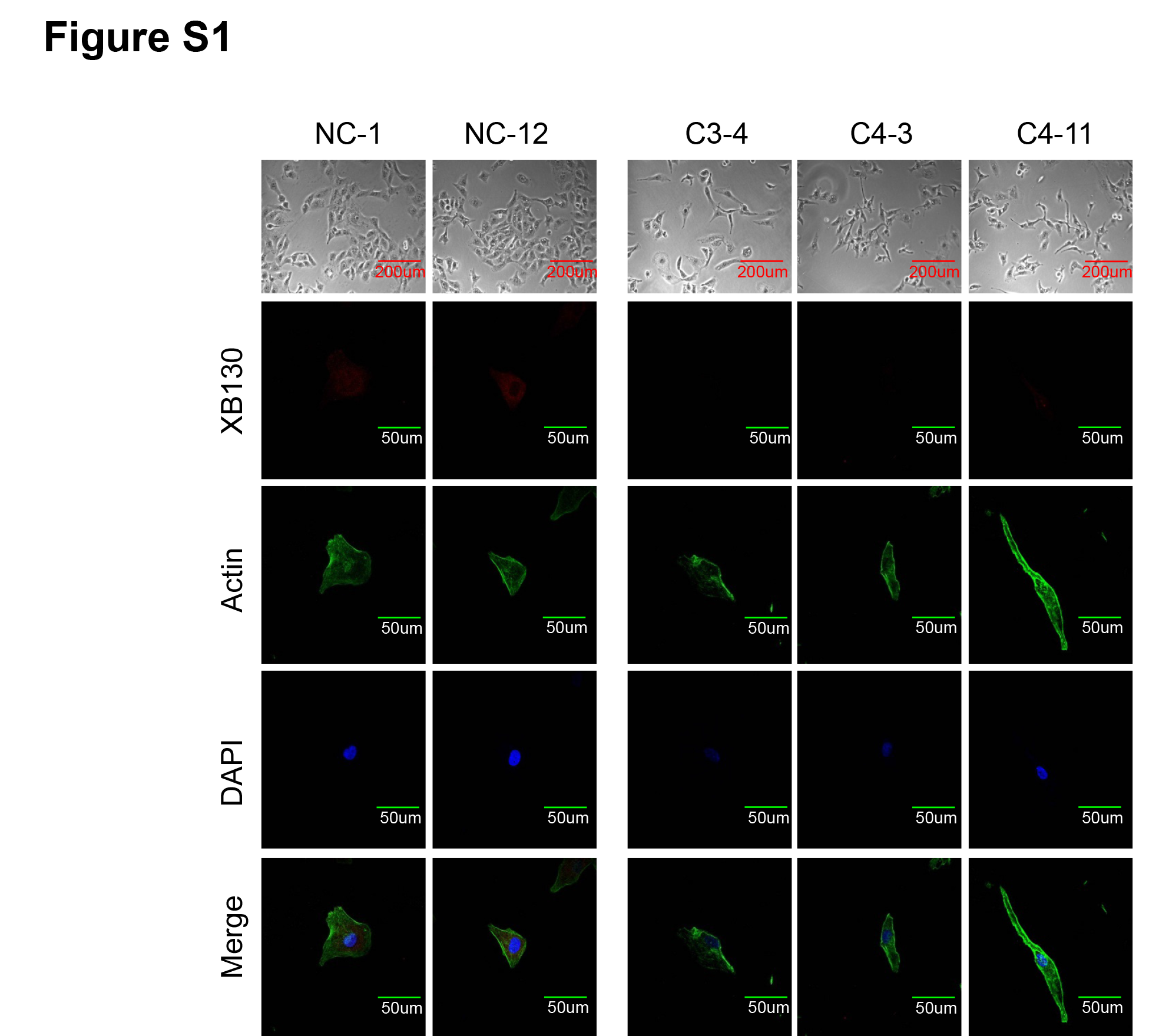

Supplement: Figure S1 — Immunofluorecence staining for XB130. As reported in Fig. 1, the other two negative control shRNA transfected WRO cell lines (NC1 and NC12) and three XB130 shRNA transfected WRO cell lines (C3-4, C4-3 and C4-11) were immune-stained with the XB130 antibody. The expression of XB130 protein significantly decreased in XB130 shRNA transfected cells. (TIF) [file pone.0059057.s001.tif]

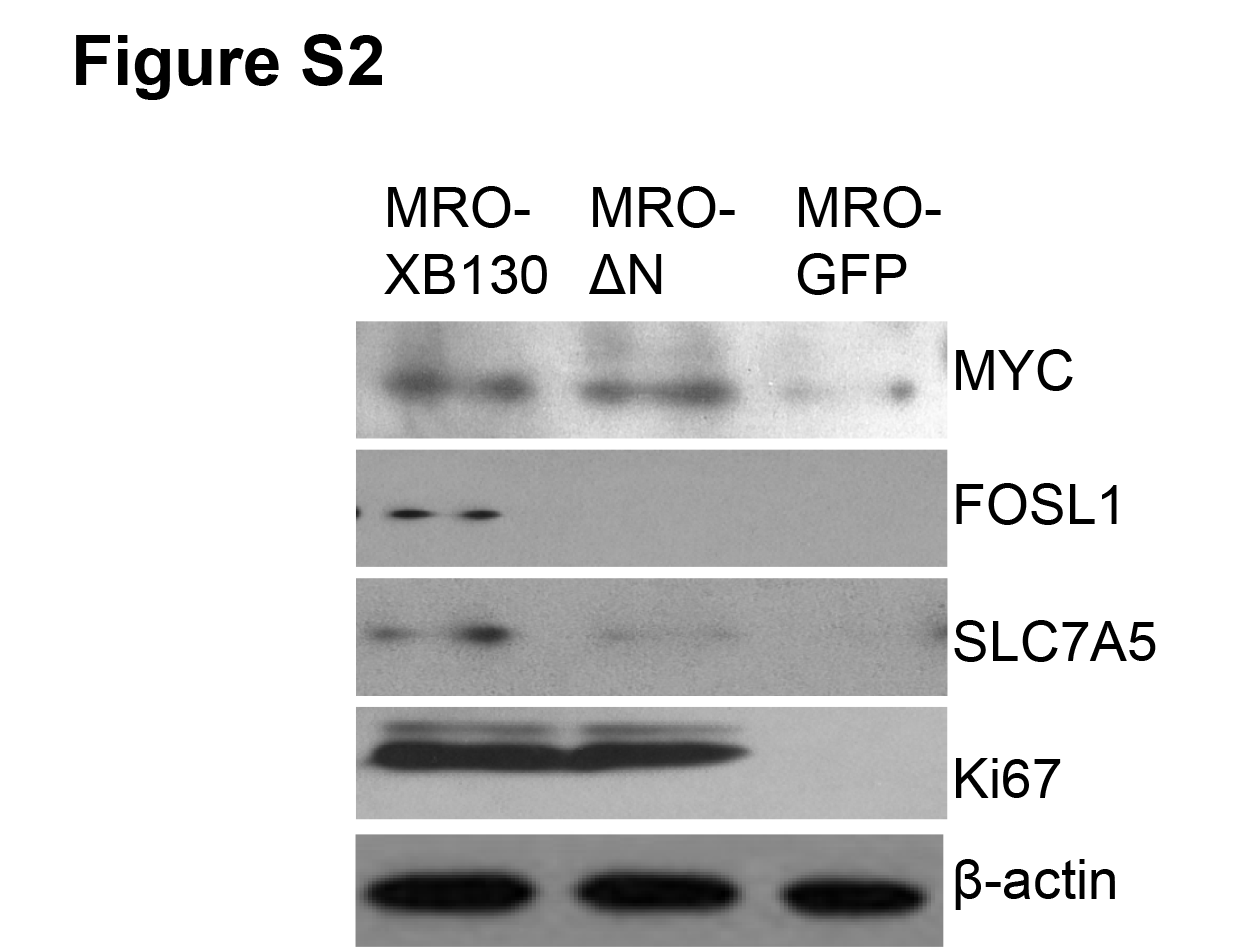

Supplement: Figure S2 — Ectopic XB130 expression in MRO cells increased levels of MYC, FOSL1, SCL7A5 and Ki67. MRO cells were transfected with GFP vector alone, or GFP-XB130, GFP-XB130ΔN (XB130 N-terminus deletion mutant). GFP positive cells were collected by FACS. Western blotting revealed higher levels of MYC, FOSL1, SCL7A5 and Ki67, in comparison to GFP-alone transfected cells. The MYC and Ki67 levels in GFP-XB130ΔN transfected cells were also higher than that in GFP-alone cells. (TIF) [file pone.0059057.s002.tif]
