# Supplementary material for: XB130, a New Adaptor Protein, Regulates Expression of Tumor Suppressive MicroRNAs in Cancer Cells
Source: PLoS One. 2013 Mar 19;8(3):e59057. doi: 10.1371/journal.pone.0059057 (PMC3602428; doi:10.1371/journal.pone.0059057)
Supplement: Supplementary file 2 [file pone.0059057.s003.doc]

Table S1

significantly downregulated miRNAs in XB130 shRNA Transfected Cells

miRNA name	Fold change	p-value
hsa-miR-431*	-37.378	<0.001
hsa-miR-32*	-36.488	0.038
hsa-miR-595	-35.001	0.002
hsa-miR-3141	-30.162	<0.001
hsa-miR-3656	-20.600	0.021
hsa-miR-500a	-18.932	0.012
hsa-miR-181d	-18.713	0.025
hsa-miR-892b	-18.614	0.031
hsa-miR-3911	-15.171	<0.001
hsa-miR-3156	-11.252	0.046
hsa-miR-23a*	-9.703	0.001
hsa-miR-338-5p	-9.635	0.001
hsa-miR-1275	-1.717	0.018
hsa-miR-29a	-1.604	0.007
hsa-miR-183	-1.550	0.011
hsa-miR-3198	-1.541	0.001
hsa-let-7i	-1.514	0.040
hsa-miR-1305	-1.499	<0.001
hsa-miR-324-3p	-1.444	0.015
hsa-miR-100	-1.433	0.030
hsa-miR-92a	-1.380	0.023
hsa-miR-320a	-1.264	0.039

Note: Two strands come from one stem loop precursor miRNA (pre-microRNA) by dicer processing. One of them is indicated with an asterisk. For example, has-miR-191* and has-miR-191 are two sequences from the same precursor.
